# Geographic Variability of Berry Phytochemicals with Antioxidant and Antimicrobial Properties

**DOI:** 10.3390/molecules27154986

**Published:** 2022-08-05

**Authors:** Cecilia Georgescu, Adina Frum, Lidia-Ioana Virchea, Anastasiia Sumacheva, Mark Shamtsyan, Felicia-Gabriela Gligor, Neli Kinga Olah, Endre Mathe, Monica Mironescu

**Affiliations:** 1 Faculty of Agriculture Science, Food Industry and Environmental Protection, “Lucian Blaga” University of Sibiu, 550012 Sibiu, Romania; 2 Faculty of Medicine, “Lucian Blaga” University of Sibiu, 550012 Sibiu, Romania; 3 Saint Petersburg State Institute of Technology, 190013 St. Petersburg, Russia; 4 Faculty of Pharmacy, “Vasile Goldis” Western University of Arad, 310045 Arad, Romania; 5 Faculty of Agricultural and Food Sciences and Environmental Management, University of Debrecen, H-4032 Debrecen, Hungary

**Keywords:** berries, phytochemicals, flavonoids, anthocyanins, polyphenols, antioxidant activity, antimicrobial activity

## Abstract

The aim of this study was to determine the variability of several chemical compounds and the antioxidant and antimicrobial activities of eight types of berries harvested from two different geographical regions in the same year. The analyses were performed on bilberry, black currant, gooseberry, red currant, raspberry, sea buckthorn, strawberry and sour cherry, which were handpicked during the summer of 2019, in the same periods when they are typically harvested for consumer purposes. Total anthocyanins content (TAC), total flavonoids content (TFC), total polyphenolic compounds (TPC), determination of the Ferric-Reducing Antioxidant Power (FRAP), determination of the DPPH free radical scavenging assay (RSA), determination of nine phenolic compounds by HPLC-UV assay and antimicrobial activity were determined for undiluted hydroalcoholic extracts of all the studied berries. The results showed that the berries from Romania were richer in antioxidant compounds than the berries from Russia. The TPC content varied between 4.13–22.2 mg GAE/g d.w., TFC between 3.33–8.87 mg QE/g d.w. and TAC between 0.13–3.94 mg/g d.w. The highest variability was determined for TPC. Regarding the antioxidant activity assessed by FRAP assay, values were between 6.02–57.23 µmols TE/g d.w. and values for the RSA method between 18.44–83.81%. From the eight types of berries analyzed, bilberries and raspberries had the highest antioxidant activity considering both regions and both determination methods. Not only the type, but also the environmental and cultivation conditions in which the berries grow, can lead to variations in their chemical composition. The extracted polyphenolic compounds from the studied berries showed antibacterial properties on pathogens, such as *Escherichia coli*, *Bacillus subtilis* and *Staphyloccocus aureus*. The inhibitory action on *Salmonella typhi* and fungi *Candida albicans* and *Aspegillus niger* was absent to very low. The antimicrobial activity of the hydroalcoholic extracts was dependent on the provenance of the berries, too.

## 1. Introduction

Nowadays, people choose their food more and more carefully because they want to follow a healthy lifestyle. Berry fruits belong to a class of foods named “superfoods”. Beside their nutritional properties, they are rich sources of bioactive compounds [1]. They have a pleasant taste, are low energetic and contain a high amount of bioactive compounds, especially when they are consumed fresh [2].

The chemical composition of berries is complex and the bioactive compounds found in berries, individually or combined, bring a lot of benefits for human health [3]. Phenolic compounds from berries are one of the chemical classes responsible for the antioxidant and antimicrobial activities berries offer [4]. They are well known for their in vitro and in vivo antioxidant activity, due to their capacity to protect against free radical induced damage [2]. Due to their phenolic compounds contents, berries are a natural source of phytochemicals with antioxidant activity, used as an alternative to synthetic antioxidants [5]. They also have other effects, such as protection against osteoporosis, neurodegenerative and cardiovascular diseases, inflammation and diabetes [1,3,6,7]. Recent studies have even reported antineoplastic effects [3]. Some researchers have reported that consumption of berries decreases the risk of breast, ovarian and melanoma cancers in humans [8]. Ethanol extracts from different berries, rich in polyphenols, showed antimicrobial activity against bacteria [9,10] or fungi [11,12]. Investigations on polyphenols, such as gallic acid and catechin, which are naturally abundant in berries, demonstrated strong action against *Escherichia coli* [13]. Phenolic extracts from berries, such as blueberry, blackcurrant, raspberry and strawberry inhibited *Helicobater pylori* [14].

The health benefits of berries vary with the type of berry because there are some differences between their qualitative and quantitative compositions [6].

Blackcurrants (*Ribes nigrum* L.), are rich in anthocyanins, flavonoids, vitamins, organic acids, unsaturated fatty acids and polysaccharides [5,8,15]. They were reported to have in vitro antioxidant and antimicrobial activities, to be immuno-stimulating, and to have antineoplastic and anti-inflammatory properties [15]. After a single dose of blackcurrant, improvement in adaptation to darkness was observed [16]. Blackcurrant juice suppressed oral bacteria [17]. Red currants (*Ribes rubrum* L.) have antioxidant activity due to their containing vitamin C and a high amount of anthocyanins and phenolic compounds [18]. Bilberries (*Vaccinium myrtillus* L.) contain significant amounts of phenolic compounds, carotenoids, and vitamins. Bilberries have beneficial effects in the prevention of cancer, cardiovascular disease, obesity, diabetes, diseases caused by aging, urinary tract infections and periodontal disease [19], Bilberries show antimicrobial effects, too, especially against *E. coli* and *Bacillus subtilis* [20]. In order to be more accessible to consumers, bilberries were added in several foods (i.e., beer) [21,22]. Gooseberries (*Ribes grossularia* L.) are a source of phenolic compounds, such as flavonols (quercetin, kaempferol and myricetin) and phenolic acids (caffeic, coumaric, ellagic and hydroxybenzoic acids). Due to their antioxidant effect on LDL-cholesterol and anti-atherosclerotic effect, the consumption of gooseberries can prevent cardiovascular disease. Gooseberries contain organic acids (citric, malic, tartaric, succinic, fumaric, glutaric and ketoglutaric) which reduce the risk of Alzheimer disease or stroke [23]. *Staphylococcus aureus* seems to be especially sensitive to gooseberry extracts [24]. Sour cherries (*Prunus cerasus* L.) contain high amounts of phenolic compounds, especially anthocyanins (of which the most abundant is cyanidin-3-glucosyl rutinoside), hydroxycinnamic acids and flavan-3-ols. They contain more procyanidins, flavanols and flavonol glycosides than sweet cherries [25]. Sour cherries also contain melatonin. Anthocyanins from sour cherries were reported to have antioxidant, antimicrobial, neuroprotective, antineoplastic, and anti-inflammatory effects [26,27]. Organic acids from sour cherries are responsible for their sour taste and increase the secretion of digestive enzymes [26]. Raspberries (*Rubus idaeus* L.) are rich in phenolic compounds, especially anthocyanins and ellagic tannins. Raspberry extracts are included in food supplements used in upper respiratory tract infections due to their antibacterial properties and immunity stimulating effect [28]. Blackberries (*Rubus fruticosus* L.) are rich in anthocyanins and ellagitannins. They also contain vitamins (C, K, folic acid). Blackberries have antioxidant properties and protect against cardiovascular diseases, cancers and other diseases [29] and they show strong antibacterial and antifungal activity [30]. Strawberries (*Fragaria x ananassa* Duch.) contain over 40 phenolic compounds, including quercetin, cyanidin, pelargonidin, kaempferol glycosides, ellagic acid, derivatives of p-coumaric acid, and ellagitannins [5]. They are used in traditional medicine for their astringent and diuretic effects. Consumption of strawberries could reduce the risk of cardiovascular disorders because the antioxidants from their composition inhibit the oxidation of LDL-cholesterol, improve endothelial function and prevent thrombosis [2]. Moreover, strawberries have antimicrobial effects, especially against *Pseudomonas* sp. [31]. Sea buckthorn (*Hippophae rhamnoides* L.) fruits are an important source of flavonoids (rutin, quercetin, kaempferol, myricetin), vitamins (E, C, B1, B2, B9 and K), carotenoids, amino acids, proteins, and minerals (K, Ca, Fe and P). The fruits have antioxidant and antimicrobial activity and they can prevent cardiovascular diseases [32,33,34].

Among others, berries, due to their vitamins C and E content could increase the defense level of the body [35]. It was reported that cyanidin-3-arabinoside, an anthocyanin found in bilberries, gooseberries, and blackcurrants, inhibits the main protease of SARS-CoV-2. Another anthocyanin, pelargonidin-3-glucoside found in raspberries binds with spike proteins of SARS-CoV-2 and inhibits binding with the ACE2 receptors of the host cells. These molecules found in berries are not the only ones which could prevent infection with SARS-CoV-2 [36].

In this study we aimed to determine the variability of several chemical compounds and their antioxidant and antimicrobial activities from eight types of berries harvested in different geographical regions in the same year. To the best of our knowledge, this is the first comprehensive report that evaluates bioactive compounds with antioxidant and antimicrobial activities, from berries harvested from regions with different climatic conditions, namely temperate continental for the Transylvania region and humid continental for the Saint Petersburg region.

## 2. Materials and Methods

### 2.1. Sample Preparation

The selected berries were bilberry (*Vaccinium myrtillus* L.), black currant (*Ribes nigrum* L.), red currant (*Ribes rubrum* L.), raspberry (*Rubus idaeus* L.), gooseberry (*Ribes uva-crispa* L.), sea buckthorn (*Hippophae rhamnoides* L.), sour cherry (*Prunus cerasus* L.), and strawberry (*Fragaria x ananassa* Duch.). They were harvested in the summer of 2019 from unpopulated areas of Transylvania, Sibiu County, Romania (S1), and from the Saint Petersburg region, Russia (S2), at the stage of maturity, dried in airflow at 40 °C until they reached a constant mass and then ground on a domestic mill. Prior to the analysis they were stored in glass containers at room temperature, away from sunlight.

### 2.2. Chemicals

The standards of gallic acid (purity > 99%), ferulic acid (purity > 99%), syringic acid (purity > 95%), cinnamic acid (purity > 99%), caffeic acid (purity > 99%), (+)-catechin (purity > 98%), resveratrol (purity > 99%), quercetin (purity > 95%), rutin (purity > 94%) and methanol suitable for HPLC analysis (purity ≥ 99.9%) were purchased from Sigma-Aldrich (St. Louis, MO, USA). TPTZ (2,4,6-tripyridyl-s-triazine), Trolox (6-hydroxy-2,5,7,8-tetramethyl- chroman-2-carboxylic acid), DPPH (2,2-diphenyl-1-picrylhyd razyl) and Folin-Ciocalteu reagent were purchased from Sigma-Aldrich, too, and ferric chloride, sodium acetate, glacial acetic acid, hydrochloric acid, and ethanol, all analytical grade purity, were purchased from the Chemical Company. In all analyses ultrapure water was used (conductivity 0.05 µS/cm).

Amoxicillin powder (Abbott, brand name Amoxiclave) was purchased from a drug-store. Fluconazole disks (25 μg) and dehydrated cultivation media Mueller-Hinton Broth and Czapek-Dox Broth were from Thermo Scientific™ Oxoid™.

### 2.3. Extractions

To determine the content in phenolic compounds, total polyphenols and total flavonoids, 0.5 g of each sample of dried fruit powder were treated with 10 mL of solvent comprised of ethanol:water:0.12M hydrochloric acid = 70:29:1 (*V/V/V*) and put into an ultrasonic bath at 40 °C for 30 min, cooled, filtered, and brought to 10 mL in a volumetric flask by using the same solvent. For the antioxidant activity assays, the same extraction method was used, using ethanol as the solvent [37].

For the total anthocyanins assay, 0.5 g of each sample of dried fruit powder were treated with 50 mL of solvent comprised of ethanol:1.5M hydrochloric acid = 85:15 (*V/V*) and put into an ultrasonic bath at 40 °C for 30 min, left at room temperature away from sunlight for 24 h, filtered and brought to 50 mL in a volumetric flask by using the same solvent [38].

### 2.4. Analysis

#### 2.4.1. Determination of the Total Anthocyanin Content (TAC)

The extinctions of the extracted samples were recorded at λ = 535 nm by using a Shimadzu UV 1900 spectrophotometer, and the total anthocyanin amounts, expressed as mg/g plant dry weight (d.w.), were calculated by using the following formula:

(1) $$\mathrm{TAC}\;(\mathrm{mg}/g\;d.w.)=\frac{E\cdot d}{98.2},$$

where: E = extinction of the solution, d = dilution factor, and 98.2 = absorption value of the solvent ethanol:1.5 M hydrochloric acid (85:15), at λ = 535 nm [38].

#### 2.4.2. Determination of the Total Flavonoid Content (TFC)

Into a 25 mL volumetric flask, 5 mL of the sample solution, 5 mL of a 100 g/L CH_3_COONa solution and 3 mL of a 25 g/L AlCl_3_ solution were added, shaken, brought to mark with ethanol and left standing for 15 min. The extinction was determined at λ = 430 nm by using a Shimadzu UV 1900 spectrophotometer. The compensation liquid consisted of 5 mL sample solution and 8 mL purified water that were added to a 25 mL volumetric flask, that was brought to mark with ethanol [39].

The calibration curve was linear for the range of 8–40 µg quercetin/mL. The equation was y = 31.487x − 0.0974 (R^2^ = 0.999) where y = extinction at λ = 430 nm and x = concentration expressed as mg quercetin/mL, and the results were expressed as mg quercetin equivalents (QE)/g d.w.

#### 2.4.3. Determination of the Total Polyphenol Content (TPC)

An amount of 0.4 mL sample solution, 1 mL of Folin-Ciocalteu reagent, 15 mL of water and 2 mL of a 290 g/L Na_2_CO_3_ solution were added into a test tube, shaken for 10 min, and kept at 40 °C for 20 min in a water bath. After cooling, the extinction was recorded at λ = 760 nm by using a Shimadzu UV 1900 spectrophotometer [37,40].

The calibration curve was linear for the range of 0.9–4.5 µg gallic acid/mL. The equation was y = 61.525x − 0.0019 (R^2^ = 0.999) where y = extinction at λ = 760 nm and x = concentration expressed as mg gallic acid/mL, and the results were expressed as mg gallic acid equivalents (GAE)/g d.w.

#### 2.4.4. Determination of the Ferric-Reducing Antioxidant Power (FRAP)

To obtain the FRAP solution, 2.5 mL of a 10 mM TPTZ solution in 40 mM HCl, 2.5 mL of a 20 mM FeCl_3_ solution and 25 mL acetate buffer (pH = 3.6) were mixed. 0.1 mL of sample solution, 0.7 mL of water and 6 mL of FRAP solution were mixed and the mixture’s extinction was recorded at λ = 593 nm by using a Shimadzu UV 1900 spectrophotometer [41,42].

The calibration curve was linear for the range of 3–24 nmol Trolox/mL, the equation was y = 0.0419x − 0.0089 (R^2^ = 0.999) where y = extinction at λ = 593 nm and x = concentration expressed as nmol Trolox/mL, and the results were expressed as µmol Trolox equivalents (TE)/g d.w.

#### 2.4.5. Determination of the DPPH Free Radical Scavenging Assay (RSA)

A stock solution of 25 µg/mL DPPH in methanol was prepared and kept at low temperature and in the dark for 2 h before usage. Then, 970 µL of DPPH stock solution were added into 30 µL of sample solution. The absorbance was recorded at 515 nm, using a Shimadzu UV 1900 spectrophotometer and the results were expressed as (%) [43,44,45]. The calibration curve was linear for the range of DPPH concentrations of 0.25–250 µg/mL. The equation based on the calibration curve was: y = 0.0127x + 0.0036 (R^2^ = 0.999), where y = extinction at λ = 515 nm and x = concentration expressed as µg DPPH/mL.

The DPPH radical scavenging activity was determined by using the following formula:

(2) $$\mathrm{RSA}\;(\%)=\frac{C_{0}-C_{1}}{C_{0}}\;\cdot 100,$$

where: RSA = DPPH radical scavenging activity (%), C_0_ = concentration of the DPPH stock solution (µg/mL) and C_1_ = DPPH concentration in the sample (µg/mL).

#### 2.4.6. Determination of Phenolic Compounds by HPLC-UV Assay

The HPLC-UV method for the identification and quantification of phenolic compounds was employed by using several methods already conducted on plants and food supplements [37,46,47].

The analysis was carried out by using a Shimadzu SCL-40 HPLC system equipped with degasser, quaternary pump, photodiode array detector, thermostatted column oven and autosampler. The used column was Nucleosil C18 (250 mm × 4.6 mm, i.d. 5 µm). The oven temperature was 25 °C. The elution was performed by using three mobile phases: A, purified water; B, methanol; and C, purified water: acetic acid (96:4 (V/V)) in a gradient program, as follows: 15% B and 85% C at 0 min, 75% A and 25% B at 15 min, 15% A and 85% B at 20 min, 40% A and 60% B at 40 min followed by column conditioning. The flow rate was 0.5 mL/min for the first 15 min and 0.8 mL/min from minute 15 to minute 40. The injection volume was 5 µL. The detection was performed at 280 nm for gallic acid, (+)-catechin, syringic acid and cinnamic acid, 306 nm for resveratrol, 330 nm for caffeic acid and ferulic acid and 360 nm for rutin and quercetin.

#### 2.4.7. Determination of the Antimicrobial Activity against Pathogens

In this study, the used pathogens were the following Gram-positive bacteria: *Bacillus subtilis* ATCC 6051, *Bacillus cereus* ATCC 12600, and *Staphylococcus aureus* ATCC 12600. The following Gram-negative bacteria were used: *Escherichia coli* ATCC 11775 and *Salmonella typhi* ATCC 1408. The following fungal strains were used: *Aspergillus niger* ATCC 10575, *Candida albicans* ATCC 10231.

For activating the pathogen bacteria and the *C. albicans* yeast strain, pure cultures of each test organism were inoculated into sterile Mueller-Hinton liquid cultivation media for bacteria and Czapek-Dox liquid cultivation media for *C. albicans* and allowed to grow for 48 h at 37 °C. The cells biomass in the liquid suspensions was adjusted by using 0.5 McFarland standard corresponding to ~7.5 × 10^7^ colony forming units (CFU)/mL for bacteria [48] and to 1~5 × 10^6^ colony forming units (CFU)/mL) for *C. albicans* and used for inoculation [49]. For determining the antifungal action of *A. niger*, the mold was cultivated as described in [50]. Then, malt liquid broth was inoculated with the mold and cultivated for 10 days at 25 °C with agitation, and, after that, the mycelium was disintegrated by intense shaking on a magnetic stirrer and the clear liquid obtained was sterile filtered (pore size 50 µm). The suspension was used for cultivation on Petri dishes.

The antimicrobial activity of extracts isolated from berries was analyzed by adapting the Kirby-Bauer disk diffusion method [51] as follows: 1 mL of each standard pathogens culture was spread over Petri dishes containing Mueller-Hinton agar medium (for bacteria) and Czapek-Dox agar (for fungi). Sterile filter paper discs (Whatmann No. 1) of 5 mm size were applied in the middle of the dishes and loaded with 20 μL of undiluted berry hydroalcoholic extracts prepared as given in Section 2.3 for the determination of the content in phenolic compounds. No tests on different diluted berry extracts were made, because of the relatively low antimicrobial activity observed during the preliminary tests. A separate screening of the antibacterial activity of the solvents used in the extractions was carried out, too. Plates cultivated with bacteria and *C. albicans* were incubated at 37 °C for 48 h. Plates cultivated with *A. niger* were maintained for 5 days at 20 °C. Petri dish images were obtained at 5 × magnification using a stereo microscope with MicroCam 5.0 (Zeiss Stemi 508, Carl Zeiss Gottingen, Germany). The Diameter of the Inhibition Zone (DIZ) around each paper disc was determined by using the AxioVision Rel 4.8 measuring software (Carl Zeiss Microscopy GMBH). The pure solvent used for extract preparation (described in Section 2.3) was used as negative control. Amoxicillin in two concentrations, 10 μg/disc (AMX10) and 20 μg/disc (AMX20), was used as a positive control for bacteria. Fluconazole (FCZ) with 25 μg/disc was used as a positive control for *C. albicans* and *A. niger*.

### 2.5. Statistical Analysis

The results were expressed as mean and standard deviation (SD) for three determinations (*n* = 3) to verify the statistical significance between the obtained results. The evaluation was performed by using IBM SPSS Statistics version 20 software (SPSS Inc., Chicago, IL, USA). One-way analysis of variance (ANOVA), along with the post hoc Tukey’s test with the significance level of *p* < 0.05, were used for the statistical analysis.

## 3. Results and Discussion

As shown in Table 1, bilberries had the highest quantities of total polyphenols, flavonoids and anthocyanins of the fruits and berries analyzed. The S2 ones showed a slight increase in anthocyanins (3.94 mg/g d.w.) compared to the S1 ones (3.58 mg/g d.w), which presented a higher content of polyphenols and flavonoids (22.20 mg GAE/g d.w. and 8.87 mg QE/g d.w.) than S2 (21.85 mg GAE/g d.w. and 6.86 mg QE/g d.w.). The results obtained for both FRAP and RSA assays were the highest for S1 bilberries (57.23 µmols TE/g d.w. respectively 83.81%), followed closely by S2 bilberries (56.31 µmols TE/g d.w. respectively 79.40%).

Blackcurrants and raspberries had a total polyphenols content greater than 8 mg GAE/g d.w., antioxidant activities greater than 33 µmols TE/g d.w. for the FRAP assay and greater than 63% for the RSA assay. The content of total flavonoids for these berries varied between 4.36 and 5.86 mg QE/g d.w. and the content of total anthocyanins between 0.70 and 2.89 mg/g d.w.

Even though the total polyphenols content for S1 and S2 sour cherries was more than 8 mg GAE/g d.w. (8.92 respectively 8.74 mg GAE/g d.w.), the antioxidant activity was lower than for the other berries that presented similar results. Thus, the FRAP assay for sour cherry was 19.65 µmols TE/g d.w. for S2 and 20.36 µmols TE/g d.w. for S1 and the RSA was 43.16% for S2 and 46.22% for S1.

The lowest antioxidant activity was registered for S1 red currant, followed closely by S2 red currant (16.25% for RSA and 6.39 µmols Trolox/g d.w. for FRAP for S1 18.44% for RSA and 6.50 µmols Trolox/g d.w. for FRAP for S2). These results concurred with the total polyphenols assay that had values of 4.39 mg GAE/g d.w. for S1 red currant and 4.26 mg GAE/g d.w. for S2 red currant. Gooseberries presented results similar to the red currants regarding the total polyphenols content and antioxidant activity. The total polyphenols content for gooseberries was 4.82 mg GAE/g d.w. for S1 and 4.13 mg GAE/g d.w. for S2 and the antioxidant assays were 6.02 µmols Trolox/g d.w. for FRAP and 21.28% for RSA in the samples harvested from S1 and 6.14 µmols Trolox/g d.w. for FRAP and 20.91% for RSA from S2. Regarding the lowest quantities of total anthocyanins, they were registered for S2 strawberries (0.13 mg/g d.w.), S1 sea buckthorn (0.17 mg/g d.w.), S1 strawberries (0.18 mg/g d.w.) and S2 sea buckthorn (0.28 mg/g d.w.) (Table 1).

Overall, the S1 analyzed berries presented higher quantities of TPC, TFC and TAC than the S2 ones, although the differences between them were low. Some exceptions were observed for black currant, sea buckthorn and strawberries for TFC and bilberries for TAC, which presented higher amounts in the S2 samples compared to the S1 ones. By comparing each berry from the two harvesting regions against the content in TPC, TFC and TAC, the results obtained were not statistically different (*p* < 0.05), except for the TFC in bilberries.

The antioxidant activity determined had a similar trend as discussed before and most of the berries harvested from the temperate continental climate region (S1) present higher antioxidant activity that the ones harvested from the humid continental climate (S2). Thus, the results obtained for the antioxidant activity assays can be correlated with the content in TPC, TFC and TAC. Different results were reported by other studies performed on berries all over the world. Bujor et al. (2016) reported a TAC between 25.7 and 34.5 mg/g d.w. and a TPC between 34.7 and 41.9 mg GAE/g d.w. for bilberries harvested from Romania [52]. Ciulca et al. (2021) also analyzed bilberries from Romania and reported an antioxidant activity between 67.67 and 85.72% by using the RSA method [53]. Åkerström et al. (2010) reported values of TAC for Swedish bilberries between 15 to 39 mg/g d.w. and Danish ones of 17 mg/g d.w. [54]. Rieger et al. (2008) reported values of TAC of Austrian bilberries between 17 and 20 mg/g d.w. [55] and Ancillotti et al. (2016) values of 36.6 mg/g d.w. for bilberries harvested from Italy [56]. Gooseberries analyzed by Filipiak-Szok et al. (2012) were reported to possess a TPC of 1.99 mg GAE/g d.w., a TFC of 0.56 mg QE/g d.w. and, by using the FRAP method, an antioxidant activity of 29.86 µmols TE/g d.w. [57]. Pantelidis et al. (2005) reported a TPC between 10.52 and 21.16 mg GAE/g d.w. and an antioxidant activity measured by the FRAP method of between 77.7 and 145.4 µmols TE/g d.w. for red currants harvested in Greece [58]. Huang et al. (2012) analyzed strawberries harvested in China and reported a TPC of 2.72 mg/g d.w., a TFC of 7.04 mg/g d.w. and TAC of 1.16 mg/g d.w. [59]. The antioxidant activities of strawberries, raspberries, sea buckthorns and bilberries harvested from Canada were analyzed using the RSA method by Li et al. (2009) and quantities of 40.33%, 51.23%, 29.97% and 34.13% were obtained [60].

Many scientific studies determined the phytocompound content and antioxidant activity of berries all over the world and observed that the variability of compounds can be attributed to different growing habitats and pedoclimatic conditions.

Nine phenolic compounds were identified and quantified by using an HPLC-UV method, as shown in Table 2. The main compounds found in the highest amounts in the analyzed berries were quercetin for bilberries, black currants, red currants, and sour cherries, (+)-catechin for black currants, gooseberries, and sea buckthorns and caffeic acid for strawberries for both S1 and S2. Even though the compounds with the highest amounts were related to the same berries, regardless of their harvesting region, they might differ in quantities. Thus, the quercetin from S2 black currant (1160.97 µg/g d.w.) was higher than the one from the S1 region (1022.67 µg/g d.w.) and the one for S1 bilberry (483.74 µg/g d.w.) was higher than the S2 bilberry one (345.57 µg/g d.w.). (+)-Catechin from S1 black currants (275.31 µg/g d.w.) was higher than from S2 ones (203.52 µg/g d.w.), S1 gooseberries (201.64 µg/g d.w.) scored higher than the S2 ones (115.60 µg/g d.w.) and S2 sea buckthorns (309.62 µg/g d.w.) scored higher than for S1 sea buckthorns (230.67 µg/g d.w.) and it was not detected in sour cherries harvested from both regions (S1 and S2). Caffeic acid had higher quantities in S2 strawberries (269.31 µg/g d.w.) than in S1 ones (170.51 µg/g d.w.). The other analyzed berries presented quantities of the mentioned phenolic compounds below 64 µg/g d.w.

Gallic acid was determined in the highest amounts in S1 black currants (139.47 µg/g d.w.) followed by S2 bilberries (119.15 µg/g d.w.), syringic acid in S2 strawberries (209.55 µg/g d.w.) followed by S1 strawberries (135.85 µg/g d.w.), cinnamic acid for S2 bilberries (187.38 µg/g d.w.) and S1 bilberries (141.87 µg/g d.w.).

The other analyzed berries presented quantities of gallic acid, syringic acid and cinnamic acid lower than 88 µg/g d.w. Rutin was quantified at over 320 µg/g d.w. for bilberries from both regions and only S1 strawberries presented a quantity over 100 µg/g d.w. from the remaining berries.

All samples analyzed presented low quantities of resveratrol (below 22 µg/g d.w.) and ferulic acid (45 µg/g d.w.).

Most of the Romanian berries presented higher quantities of gallic acid, (+)-catechin, resveratrol, rutin and quercetin, whereas syringic acid and cinnamic presented higher quantities in the Russian berries. Caffeic acid and ferulic acid were determined to have equal amounts in both geographical regions analyzed. From the nine analyzed phytocompounds, seven of them had larger amounts in the Romanian bilberries compared to the Russian ones.

The variability of the compounds analyzed from the two studied regions was similar, and only (+)-catechin from sour cherries was not detected in both. Regarding the quantities of phytocompounds determined, the highest quantities of the compounds analyzed were determined for Romanian bilberries, gooseberries, red currants, raspberries, and sour cherries. Thus, black currants, sea buckthorns, and strawberries presented higher quantities of phenolic compounds analyzed from Russia. By comparing each berry from the two harvesting regions to each phenolic compound analyzed, the results obtained were statistically different (*p* < 0.05), except for the syringic acid in gooseberries, cinnamic acid in gooseberries and sour cherries, resveratrol in black currants and red currants, caffeic acid in red currants, ferulic acid in black currants, red currants, raspberries and strawberries, rutin in red currants and quercetin in red currants and strawberries. Li et al. (2009) analyzed several phenolic compounds from strawberries, raspberries, bilberries, and sea buckthorns harvested from Canada. The gallic acid was 212 µg/g d.w. for strawberries, 1129 µg/g d.w. for raspberries, 190 µg/g d.w. for bilberries and 42 µg/g d.w. for sea buckthorn. The reported caffeic acid was 24 µg/g d.w. for strawberries, 34 µg/g d.w. for raspberries, 1473 µg/g d.w. for bilberries and 10 µg/g d.w. for sea buckthorn. The syringic acid was determined only for bilberries at a quantity of 286 µg/g d.w. and the ferulic acid was quantified at 14 µg/g d.w. for strawberries, 35 µg/g d.w. for raspberries, 41 µg/g d.w. for bilberries and 15 µg/g d.w. for sea buckthorn [60]. A study performed by Ancillotii et al. (2016) on bilberries harvested from Italy, reported 33.3 µg/g d.w. gallic acid, 0.44 µg/g d.w. ferulic acid, 3.1 µg/g d.w. caffeic acid, 13.9 µg/g d.w. catechin and 2.2 µg/g d.w. quercetin [56]. Filipiak-Szok et al. (2012) analyzed phenolic compounds from gooseberries and reported a rutin content of 7.9 µg/g d.w., gallic acid of 38.7 µg/g d.w., caffeic acid 7.2 µg/g d.w., ferulic acid 2.7 µg/g d.w. and quercetin 2.7 µg/g d.w. [57]. Hajazimi et al. (2016) harvested bilberries and sea buckthorns from Sweden and analyzed several phenolic compounds. They reported 68 µg/g d.w. gallic acid for bilberries, 830 µg/g d.w. caffeic acid for bilberries and 56 µg/g d.w. for sea buckthorns, ferulic acid 148 µg/g d.w. for bilberries and 48 µg/g d.w. for sea buckthorns and quercetin 439 µg/g d.w. for bilberries and 212 µg/g d.w. for sea buckthorns [61]. Berk et al. (2020) analyzed red currantssssss from Turkey and found gallic acid 8.05 µg/g d.w., catechin 132.75 µg/g d.w., syringica acid 5.15 µg/g d.w., ferulic acid 13.95 µg/g d.w., rutin 99.30 µg/g d.w. and quercetin 6.30 µg/g d.w. [62].

**Figure 1:**
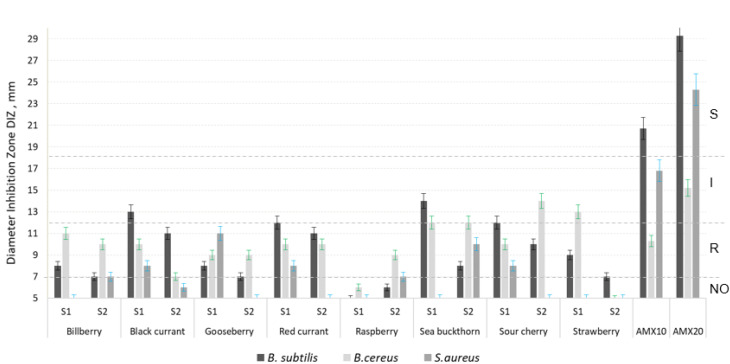
Diameter of the Inhibition Zone (DIZ) of the phenolic compounds obtained from S1 and S2 berries, against three Gram-positive pathogenic strains: *Bacillus subtilis*, *Bacillus cereus* and *Staphylococcus aureus*, together with positive controls: amoxicillin 10 μg/disc (AMX10) and amoxicillin 20 μg/disc (AMX20). NO: no action (DIZ < 7 mm); R: resistant (DIZ7-12 mm); I: intermediate (DIZ between 12–18 mm); S: susceptible (S) (DIZ > 18 mm); S1: berries harvested from Romania; S2: berries harvested from Russia. The results are expressed as mean ± SD, *n* = 3.

**Figure 2:**
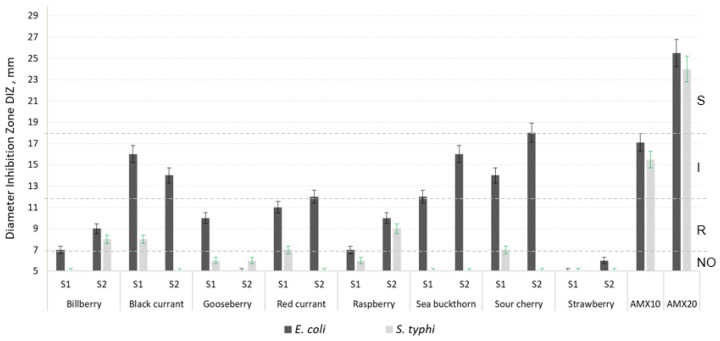
Diameter of the Inhibition Zone (DIZ) of the phenolic compounds obtained from S1 and S2 berries, against two Gram-negative pathogenic strains: *Escherichia coli* and *Salmonella typhi*, together with positive controls: amoxicillin 10 μg/disc (AMX10) and amoxicillin 20 μg/disc (AMX20). NO: no action (DIZ < 7 mm); R: resistant (DIZ7-12 mm); I: intermediate (DIZ between 12–18 mm); S: susceptible (S) (DIZ > 18 mm); S1: berries harvested from Romania; S2: berries harvested from Russia. The results are expressed as mean ± percentage deviation, *n* = 3.

**Figure 3:**
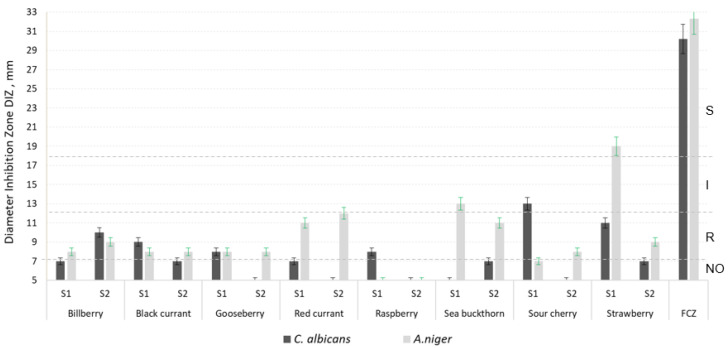
Diameter of the Inhibition Zone (DIZ) of the phenolic compounds obtained from S1 and S2 berries, against two fungal pathogenic strain: *Candida albicans* and *Aspergillus niger*, together with positive control fluconazole (FCZ) with 25 μg/disc). NO: no action (DIZ < 7 mm); R: resistant (DIZ7-12 mm); I: intermediate (DIZ between 12–18 mm); S: susceptible (S) (DIZ > 18 mm); S1: berries harvested from Romania; S2: berries harvested from Russia. The results are expressed as mean ± percentage deviation, *n* = 3. Although antimicrobial activities of obtained polyphenols from berries were generally much lower than the antimicrobial effects of antibiotics, some of them demonstrate significant antibacterial effects during studies in-vitro, comparable to the effect of an antibiotic at a low dose.

Figure 1, Figure 2 and Figure 3 show the antimicrobial activity of the phenolic compounds obtained from berries harvested from the two analyzed regions, against the seven pathogenic microorganisms considered here. The values for the diameter of inhibition zone (DIZ) presented in all figures starts from 5 mm (which was the diameter of the sterile paper disc impregned with berry extract). A diameter of inhibition zone higher than 5 indicated inhibitive action against a microorganism.

According to standards and guides, a microorganism can be classified as sensitive, intermediate or resistant to a given antimicrobial agent based on the DIZ [63,64]. Such regulations are available for antibiotics, but no rules are established yet for other antimicrobial compounds. We used a four category classification system by using the indications of [65]: no action (NO) (<7 mm), resistant (R) (7–12 mm), intermediate (I) (12–18 mm), and susceptible (S) (>18 mm). As Figure 1, Figure 2 and Figure 3 show, most of the microorganisms presented resistance or intermediate activity against the berry extracts obtained from both regions, S1 and S2. In the cases of raspberry and strawberry extracts, the activity against the Gram-positive pathogenic bacteria was very low to absent (Figure 1). The strawberry extract had no action against the Gram-negative strains (Figure 2), whereas the raspberry extract showed zero to very low antifungal effect (Figure 3).

The most sensitive microorganism to berries was *E. coli* (Figure 2), especially the S2 sour cherry extract, followed by the S2 sea buckthorn extract and the S1 black currant extract which showed high to medium antimicrobial action against the *E. coli* strain. Literature shows that tannins (especially the pro-anthocyanidins) from berries have good preventive action against urinary tract infections, caused especially by *E. coli* [66,67] and this could be a very good explanation for the efficiency of all berry extracts on this microorganism. *S. typhi* was the most resistant microorganism to berries; only the S2 raspberry extract showed a weak effect against the *S. typhi* strain (Figure 2).

As the results reveal, S1 sea buckthorn extract was the most efficient against *B. subtilis*. S2 sour cherry showed the best inhibition on three microorganisms: *B. cereus*, *E. coli* and *C. albicans*. *S. aureus* was the most responsive to the S1 gooseberry extract. S2 raspberry showed the highest efficiency against *S. typhi* and S1 [ ]: S2 bilberry, S1 gooseberry and S1 red currant.

Most of the Romanian berries presented higher antimicrobial efficiency against *B. subtilis*, *B. cereus*, *S. aureus*, *S. typhi* and *C. albicans* and the Russian ones against *E. coli* and *A. niger*.

The Committee on Herbal Medicinal Products (2015) [68] recommends bilberry, dried or as tea or extracts, to be especially used in diarrhea-related diseases, in which *E. coli* is frequently involved [69]. Some studies indicate a good antimicrobial activity of aqueous and ethanolic extracts of bilberry fruits, especially against different strains of *E. coli*, with Minimal Inhibitory Concentration (MIC) between 5 and 40 mg/mL [70], or those of methanolic extracts (50 µL of extract) on many Gram-negative test cultures (including *E. coli*) and Gram-positive microorganisms (as *S. aureus* and *B. subtilis* spores and vegetative cells) [71]. Bilberry extract obtained by us seemed to have a different behavior, with absent to low antimicrobial activity against all classes of analyzed pathogens (Figure 1, Figure 2 and Figure 3), compared to the literature and to the other extracts analyzed here. These results are in accordance with other studies revealing low antimicrobial action of bilberry extracts on the bacteria *Staphylococcus epidermidis*, *Bacillus subtilis* and *Escherichia coli* and on the pathogenic yeast *Candida albicans* [14,72]. As different studies suggest, not only phenolics could be responsible for the inhibition of bacterial pathogens; other compounds, such as organic acids or terpenes (not investigated here), could influence the growth of the microorganisms growth, too [66,70,73]. Small differences between S1 and S2 were found; except for the action on *B. cereus*. The S2 samples showed better antimicrobial power than S1, even though the content of phenolic compounds was generally higher in the S1 bilberry (Table 2).

The black currant extract was much more efficient against *B. subtilis* (Figure 1) and *E. coli* (Figure 2) than that from bilberry, with a significant increased action in S1 samples compared to S2. Paunović et al. [74] indicated the high antimicrobial action of black currant extracts, too, with *E. coli* as the most sensitive. As other research shows, phenolic acids (cinnamic acid, 3-coumaric acid, caffeic acid and ferulic acid) seem to be responsible for the strong activity against Gram-negative bacteria [75]. Their presence in higher quantities in the S1 samples than in S2 samples (Table 2) could explain the strongest antimicrobial activity of the extracts from S1. In the case of fungi *A. niger* and *C. albicans*, our research showed weaker action of the black currant extract on them (Figure 3), compared to the bacteria. Results from other authors indicate strong action of the black currant extract against the fungal strains *A. niger* and *C. albicans*, in a concentration of 38.2 µg/mL to 56.9 µg/mL, depending on the cultivar [74], contrary to our result. On the other hand, weak activity against *C. albicans*, where no growth inhibition at the highest concentration tested of 1:4 powder extract: water or 1% methanol was detected was reported in [12], in accordance with our results.

The bacterial strains investigated by us were resistant to the gooseberry extract (Figure 1 and Figure 2) and the antifungal activity was in the low range (Figure 3), in accordance with Krisch et al., [12] who reported a low to absent anticandidal activity of the methanolic gooseberry extracts (with no growth inhibition at the highest concentration tested of 1:4 powder extract:water or 1% methanol). Bendokas et al. indicated B. cereus, *E. coli* and S. aureus as being intermediate to susceptible to one type of Ribes uva-crispa (5 mL of 0.1, 0.5 and 1% methanolic extracts) [76].

With some exceptions, the red currant extracts from both S1 and S2 origins behaved similar against the microorganisms studied; all pathogenic strains were resistant to red currant ethanolic extract. Similar results were reported by Aly et al. by testing an extract of 0.1 mL on the Gram-positive bacteria S. aureus, Streptococcus faecalis and B. cereus, on the Gram-negative strains *E. coli* and Ps. aeruginosa, and on the molds *A. niger*, A. flavus and Penicillium and Rhyzopus species. They obtained inhibition areas between 8 and 13 mm [77].

The raspberry hydroalcoholic extract from both regions analyzed showed none to low antimicrobial activity against the Gram-positive bacteria (Figure 1) and fungi (Figure 3). Some other studies showed relatively good antimicrobial action of extracts prepared in a similar way against a *B. subtilis* strain, indicating the importance of the plant variety as one of the main differences [28]. Aly et al. obtained better results than us by using raspberry ethanolic extracts (0.1 mL) on pathogens [77].

Sandulachi et al. indicated very good results by using sea buckthorn in different forms on *S. aureus*, followed by *B. subtilis*, *S. typhimurium* and *E. coli*. *C. albicans* was resistant to sea buckthorn. They showed the importance of the form in which the fruit is consumed (as fruit puree, extracts with different solvents or dried), together with the importance of species and concentration [78]. Only in the cases of *B. subtilis* (on which S1 sea buckthorn showed intermediate efficiency) and *E. coli* (intermediate to S2 sea buckthorn) did we obtain comparable results with those from the literature.

The sour cherry extract behaved similar to the red currant extract (Figure 1, Figure 2 and Figure 3), even though their total polyphenols, flavonoids, and anthocyanins contents, and their antioxidant activity were different (Table 1 and Table 2). The microorganisms were resistant to both S1 and S2 sour cherry extracts, with the notable exception of *E. coli* (which was intermediate until sensitive to the extract, Figure 2). Other results showed that juice and extract obtained from *Prunus cerasus* exhibited antibacterial activity (especially the juice in a concentration of 25 mg/mL on *E. coli* and the extract in a concentration of 20 mg/mL on *S. typhymurium*), but had no antifungal activity against pathogens [27]. It was very difficult to correlate the content in phenolic compounds with the antimicrobial action. For example, catechin, which was present in very large amounts in S1 and S2 sea buckthorn, was indicated by the literature to have a strong action on *Bacillus cereus* [79]. However, catechin was not detected in our sour cherry samples (as Figure 2 presents), indicating middle to high inhibitory activity on *B. cereus*.

Strawberry extracts are indicated by the literature as being very efficient against bacteria, such as *E. coli* (in quantities varying from 0.8 to 7 mL, or 15 µL of diluted extract in sterile distilled water (0.1 *w/v*) [75,80]) and showing no action on fungi, such as *C. albicans* [80]. Their results highly contrast with ours, which showed resistance to sensitivity of fungi to the S1 and S2 strawberry (Figure 3), and no action of both extracts on *E. coli* (Figure 2). The extraction methods were different from ours; acetone was used by [75] and methanol by [80] and perhaps the compounds extracted were different, giving the different antimicrobial action to that of ours.

Bendokas et al. concluded that there is no correlation between the anthocyanins in berry extracts and their antimicrobial capacity; they indicated that extracts with a lower anthocyanin–to-phenolics ratio more effectively inhibited bacterial growth [76]. We can add that the berries’ antimicrobial action was different and was strongly dependent on the provenance of the phenolic compounds (berry type and location) and on the pathogenic strain on which their action was tested. Such results are very much dependent on the method used at the extraction of valuable compounds, too. It is difficult to compare the values with those given by other authors, due to the lack of standardization.

## 4. Conclusions

All eight types of berries analyzed in this study (bilberry, black currant, red currant, raspberry, gooseberry, sea buckthorn, sour cherry, and strawberry) present a high quantity of compounds that possess antioxidant and antimicrobial activities. For this reason, these berries can be used as a possible source of pigments, phenolics, nutraceuticals and flavonoids in nutritional studies. Results indicated a different phytochemical composition depending on their place of origin. To our knowledge, the variation of biologically active compounds, antioxidant activity, and antimicrobial properties in wild berries have not been previously systematically investigated. The development of dietary supplements using berries that have phytocompounds with antioxidant and antimicrobial properties could be considered in order to use them for the prevention of chronic diseases accompanied by an increase in free radical scavenging reactions. In this regard, polyphenols, which occupy a leading place among exogenous natural antioxidants, are of great interest. Despite the large amount of research carried out in recent years, there is still no clear understanding of the antimicrobial mechanisms of action of these substances. It should be admitted that this area of science is at the stage of accumulating facts, and the creation of a unified theoretical basis explaining the action of polyphenols remains a matter for the future.

## Figures and Tables

**Table 1:** The content in total polyphenols, flavonoids, anthocyanins, and antioxidant activity for berries.

Sample	TPC mg GAE/g d.w.	TFC mg QE/g d.w.	TAC mg/g d.w.	FRAP µmols TE/g d.w.	RSA %
S1					
Bilberry	22.20 ± 0.33 ^a^	8.87 ± 0.23 ^a^	3.58 ± 0.37 ^a,b^	57.23 ± 0.22 ^a^	83.81 ± 0.64 ^a^
Black currant	10.23 ± 0.72 ^c^	4.72 ± 0.35 ^d,f^	2.89 ± 0.63 ^b,c^	36.51 ± 0.69 ^d^	77.38 ± 0.48^c^
Gooseberry	4.82 ± 0.75 ^g^	3.33 ± 0.20 ^h,i^	0.25 ± 0.16 ^d^	6.02 ± 0.42 ^j^	21.28 ± 0.70 ^j^
Red currant	4.39 ± 0.50 ^g^	3.55 ± 0.42 ^e,f,g,h,i^	0.42 ± 0.31 ^d^	6.39 ± 0.41 ^j^	16.25 ± 0.25 ^l^
Raspberry	12.72 ± 0.74 ^b^	4.61 ± 0.37 ^d,h^	0.88 ± 0.35 ^d^	40.15 ± 0.39 ^b^	70.65 ± 0.48 ^d^
Sea buckthorn	7.40 ± 0.16 ^e,f^	4.36 ± 0.45 ^e,f,g,h,i^	0.17 ± 0.07 ^d^	18.38 ± 0.33 ^g,h^	34.62 ± 0.38 ^h^
Sour cherry	8.92 ± 0.15 ^d^	7.40 ± 0.18^b^	0.20 ± 0.10 ^d^	20.36 ± 0.18 ^f^	46.22 ± 0.38 ^f^
Strawberry	7.79 ± 0.22 ^d,e^	4.05 ± 0.51 ^e,f,g,h,i^	0.18 ± 0.10 ^d^	12.83 ± 0.78 ^i^	33.84 ± 0.62 ^h^
S2					
Bilberry	21.85 ± 0.56 ^a^	6.86 ± 0.81 ^b,c^	3.94 ± 0.32 ^a^	56.31 ± 0.68 ^a^	79.40 ± 0.91 ^b^
Black currant	8.94 ± 0.12 ^c,d^	5.86 ± 0.65 ^c,d^	2.49 ± 0.24 ^c^	33.30 ± 0.25 ^e^	69.55 ± 0.73 ^d^
Gooseberry	4.13 ± 0.39 ^g^	3.63 ± 0.19 ^e,f,g,h,i^	0.17 ± 0.08 ^d^	6.14 ± 0.39 ^j^	20.91 ± 0.95 ^j^
Red currant	4.26 ± 0.15 ^g^	3.54 ± 0.19 ^e,f,g,h,i^	0.26 ± 0.12 ^d^	6.50 ± 0.19 ^j^	18.44 ± 0.75 ^k^
Raspberry	12.42 ± 0.43 ^b^	4.36 ± 0.46 ^e,f,g,h,i^	0.70 ± 0.14 ^d^	37.90 ± 0.68 ^c^	63.99 ± 0.39 ^e^
Sea buckthorn	7.16 ± 0.38 ^e,f^	4.73 ± 0.39 ^d,e^	0.28 ± 0.17 ^d^	17.59 ± 0.39 ^h^	32.60 ± 0.90 ^h^
Sour cherry	8.74 ± 0.11 ^d^	6.25 ± 0.57 ^b,c^	0.35 ± 0.08 ^d^	19.65 ± 0.12 ^f,g^	43.16 ± 0.31 ^g^
Strawberry	7.75 ± 0.12 ^d,f^	4.72 ± 0.25 ^d,g^	0.13 ± 0.03 ^d^	12.61 ± 0.28 ^i^	30.23 ± 0.81 ^i^

TPC: total polyphenol content; TFC: total flavonoid content; TAC: total anthocyanin content; FRAP: ferric-reducing antioxidant power; RSA: radical scavenging assay; S1: berries harvested from Romania; S2: berries harvested from Russia. The results are expressed as mean ± standard deviation (SD), *n* = 3. The results in the same column followed by the same letters are not significantly different (*p* < 0.05).

**Table 2:** HPLC-UV analysis of phenolic compounds content in the analyzed berries.

Concentration of Phenolic Compound (µg/g d.w.)									
	Gallic Acid	(+)-Catechin	Syringic Acid	Cinnamic Acid	Resveratrol	Caffeic Acid	Ferulic Acid	Rutin	Quercetin
S1									
Bilberry	92.48 ± 0.97 ^d^	139.34 ± 1.17 ^e^	46.92 ± 1.48 ^e^	141.87 ± 1.74 ^b^	9.86 ± 0.76 ^d^	19.33 ± 0.71 ^f^	4.47 ± 0.48 ^g,i^	378.60 ± 0.93 ^a^	483.74 ± 1.55 ^c^
Black currant	139.47 ± 0.65 ^a^	275.31 ± 1.67 ^b^	41.56 ± 1.10 ^f^	50.97 ± 1.25 ^e^	4.85 ± 0.11 ^e,g^	33.50 ± 1.13 ^e^	13.43 ± 1.00 ^e^	64.88 ± 0.93 ^f^	1022.67 ± 1.27 ^b^
Gooseberry	41.63 ± 0.68 ^h^	201.64 ± 0.73 ^d^	6.71 ± 0.38 ^i,j^	16.84 ± 0.42 ^g^	18.34 ± 0.34 ^b^	10.69 ± 0.95 ^h^	14.63 ± 1.15 ^e^	15.87 ± 0.50 ^i^	59.32 ± 1.37 ^j^
Red currant	72.03 ± 0.47 ^f^	65.48 ± 1.30 ^i^	5.45 ± 0.57 ^j,l^	5.27 ± 0.25 ^i,k^	2.25 ± 0.30 ^h,i^	10.24 ± 1.02 ^h^	1.51 ± 0.53 ^k^	11.55 ± 0.85 ^j^	129.89 ± 1.70 ^g^
Raspberry	18.87 ± 1.27 ^k^	62.56 ± 0.84 ^i^	3.67 ± 0.38 ^k,l^	1.58 ± 0.43 ^l^	5.62 ± 0.27 ^e,f^	14.55 ± 0.60 ^g^	4.14 ± 0.41 ^g,j^	45.62 ± 1.25 ^g^	53.83 ± 1.54 ^k^
Sea buckthorn	79.36 ± 1.29 ^e^	230.67 ± 0.98 ^c^	24.67 ± 0.83 ^h^	20.37 ± 0.97 ^f^	17.76 ± 0.57 ^b^	33.17 ± 0.27 ^e^	18.20 ± 0.44 ^d^	45.26 ± 0.42 ^g^	86.53 ± 0.57 ^i^
Sour cherry	27.80 ± 1.16 ^j^	n.d.	27.37 ± 1.62 ^h^	7.71 ± 0.35 ^h,i^	13.41 ± 0.58 ^c^	62.29 ± 1.00 ^c^	44.74 ± 0.56 ^a^	43.65 ± 0.71 ^g^	266.22 ± 1.03 ^e^
Strawberry	27.20 ± 1.30 ^j^	36.45 ± 1.21 ^l^	135.85 ± 1.50 ^b^	52.50 ± 1.46 ^e^	18.89 ± 0.27 ^b^	170.51 ± 1.11 ^b^	4.52 ± 0.21 ^g,h^	119.44 ± 0.61 ^c^	6.87 ± 0.23 ^m^
S2									
Bilberry	119.15 ± 1.58 ^b^	87.16 ± 1.76 ^g^	34.84 ± 0.67 ^g^	187.38 ± 1.04 ^a^	6.08 ± 0.34 ^e^	11.08 ± 0.16 ^h^	2.00 ± 0.17 ^k^	322.61 ± 1.72 ^b^	345.57 ± 0.84 ^d^
Black currant	93.43 ± 1.25 ^c,d^	203.52 ± 1.96 ^d^	63.30 ± 1.09 ^d^	67.33 ± 1.10 ^d^	3.43 ± 0.52 ^g,i^	64.62 ± 0.67 ^c^	14.57 ± 1.13 ^e^	45.25 ± 0.45 ^g^	1160.97 ± 1.03 ^a^
Gooseberry	31.59 ± 1.35 ^i^	115.60 ± 0.83 ^f^	6.02 ± 0.04 ^j,k^	15.68 ± 0.57 ^g^	14.17 ± 1.06 ^c^	14.01 ± 0.26 ^g^	10.44 ± 0.79 ^f^	9.74 ± 0.54 ^j^	46.28 ± 0.80 ^l^
Red currant	63.28 ± 1.17 ^g^	41.91 ± 0.69 ^k^	9.01 ± 0.26 ^i^	6.40 ± 0.37 ^i,j^	1.79 ± 0.55 ^i^	9.09 ± 0.54 ^h^	1.94 ± 0.65 ^k^	10.91 ± 0.28 ^j^	126.71 ± 1.12 ^g^
Raspberry	6.23 ± 0.36 ^l^	77.89 ± 0.67 ^h^	8.91 ± 0.27 ^i^	4.85 ± 0.25 ^j,k^	3.51 ± 0.43 ^g,h,i^	10.43 ± 0.99 ^h^	2.43 ± 0.23 ^h,i,j,k^	37.83 ± 0.32 ^h^	48.31 ± 1.15 ^l^
Sea buckthorn	96.72 ± 1.07 ^c^	309.62 ± 0.71 ^a^	36.94 ± 1.01 ^g^	15.50 ± 1.03 ^g^	21.90 ± 0.77 ^a^	46.55 ± 1.18 ^d^	20.97 ± 0.66 ^c^	66.73 ± 1.23 ^e,f^	122.39 ± 1.30 ^h^
Sour cherry	9.06 ± 0.50 ^l^	n.d.	71.64 ± 0.93 ^c^	9.91 ± 0.28 ^h^	4.09 ± 0.16 ^f,g,i^	21.24 ± 0.74 ^f^	24.34 ± 1.14 ^b^	68.93 ± 1.37 ^e^	142.51 ± 1.53 ^f^
Strawberry	17.73 ± 1.31 ^k^	49.02 ± 1.31 ^j^	209.55 ± 0.93 ^a^	87.62 ± 1.01 ^c^	14.69 ± 0.90 ^c^	269.31 ± 1.52 ^a^	4.78 ± 0.37 ^g^	88.59 ± 1.16 ^d^	8.65 ± 1.03 ^m^

S1: berries harvested from Romania; S2: berries harvested from Russia. The results are expressed as mean ± standard deviation (SD), *n* = 3. The results in the same column followed by the same letters are not significantly different (*p* < 0.05). n.d. = not detected.

## Data Availability

Data is contained within the article.
